# Etymologia: Tetanus

**DOI:** 10.3201/eid1810.ET1810

**Published:** 2012-10

**Keywords:** etymologia, tetanus, Clostridium tetani, Aretaeus

## Tetanus [tet′ə-nəs]

From the Greek *tetanos* (“tension,” from *teinein*, “to stretch”), an often fatal infectious disease caused by the anaerobic bacillus *Clostridium tetani*. Tetanus was well known to the ancients; Greek physician Aretaeus wrote in the first century ad, “Tetanus in all its varieties, is a spasm of an exceedingly painful nature, very swift to prove fatal, but neither easy to be removed.” Active immunization with tetanus toxoid was described in 1890, but cases continue to be reported (275 in the United States from 2001 through 2010), almost exclusively in persons who were never vaccinated or had not received a booster immunization in the previous 10 years. In developing countries, neonatal tetanus—when infants are infected through nonsterile delivery—is a major contributor to infant mortality. Worldwide, an estimated 59,000 infants died of neonatal tetanus in 2008.
